# Xenogeneic matrix for basal cell carcinoma reconstruction: a case report

**DOI:** 10.1093/jscr/rjae396

**Published:** 2024-06-03

**Authors:** Helmut Hildebrandt, Peer W Kämmerer, Diana Heimes, Amely Hartmann

**Affiliations:** Department of Oral and Maxillofacial Surgery, Private Practice, Leher Heerstraße 77, 28359 Bremen, Germany; Department of Oral and Maxillofacial Surgery/Plastic Surgery, University of Mainz, Augustusplatz 2 55131 Mainz, Germany; Department of Oral and Maxillofacial Surgery, Private Practice, Leher Heerstraße 77, 28359 Bremen, Germany; Department of Oral and Maxillofacial Surgery/Plastic Surgery, University of Mainz, Augustusplatz 2 55131 Mainz, Germany; Department of Oral and Maxillofacial Surgery/Plastic Surgery, University of Mainz, Augustusplatz 2 55131 Mainz, Germany; Department of Oral and Maxillofacial Surgery, Private Practice, Volmarstr.8, 70794 Filderstadt, Germany

**Keywords:** matrix, basal cell carcinoma, neoplasia, I-PRF, quality of life, xenogeneic

## Abstract

This case report introduces an innovative approach for tissue regeneration post-total excision of basal cell carcinoma utilizing a xenogeneic collagen matrix coupled with injectable platelet-rich fibrin. The clinical outcome underscores the efficacy and predictability of this protocol in soft tissue regeneration. While further investigation on a larger patient cohort is warranted to fully elucidate its effects and advantages, this technique holds promise in streamlining surgical procedures following excision of extraoral neoplasms. Notably, its simple handling, minimal resource requirements, and potential to mitigate donor site morbidity and patient comorbidities post-surgery signify its value in clinical practice.

## Introduction

The incidence of cutaneous malignant neoplasms is on the rise, attributed to factors such as an aging population and increased ultraviolet (UV) radiation exposure. Basal cell carcinoma (BCC) stands as the most prevalent malignancy affecting fair-skinned individuals globally. Notable predisposing factors include UV exposure, particularly UVB, Northern European descent, childhood sunburn history and tanning bed usage, alongside others such as arsenic exposure, immunosuppression, and transplant or HIV patients [1, 2]. Predominantly affecting men and the elderly, early detection through clinical examinations, dermoscopy and advanced imaging techniques like reflectance confocal microscopy is crucial [3].

While rare, advanced BCC forms with local invasion, including intracranial invasion, are reported, with larger tumors presenting a heightened risk of metastasis, local recurrence and mortality [4, 5]. Hedgehog pathway inhibitors and systemic agents constitute standard treatments for metastatic disease, with surgery remaining the cornerstone of curative treatment, tailored to individual patient and tumor characteristics [1]. Mohs micrographic surgery is advocated for high-risk lesions, while lower-risk cases may benefit from topical therapies or less invasive surgical methods such as laser surgery or curettage [6]. The choice of wound closure technique is influenced by the tumor size, location and patient-specific factors, ranging from primary closure for smaller lesions to skin grafts or flaps for larger defects. However, challenges persist in donor-site availability and patient comorbidities, necessitating innovative approaches. This case report presents the utilization of a bioactivated xenogeneic collagen matrix for facial defect reconstruction following limited BCC excision, highlighting its potential as a solution to overcome donor-site limitations.

## Materials and methods

**Figure 1:**
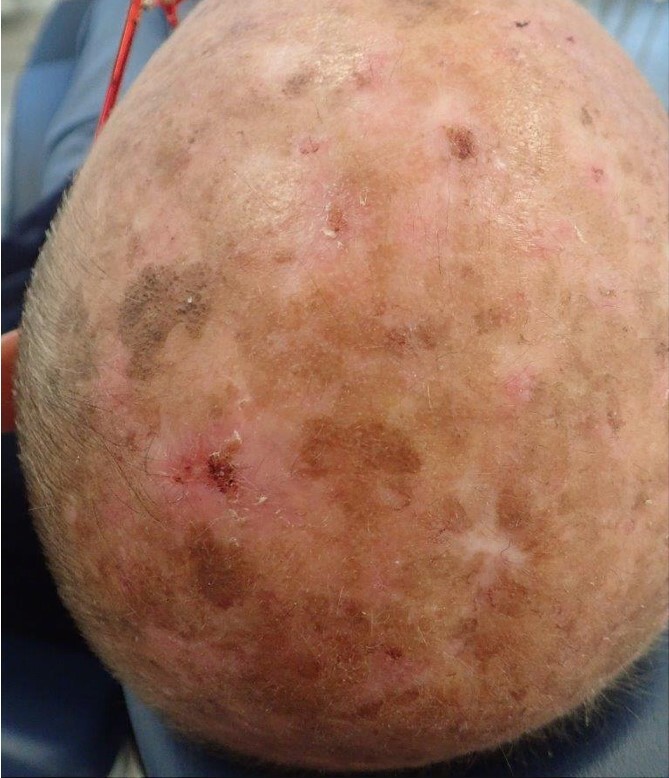
Clinical appearance of the lesion(s). The wound healing is challenging due to exposure to environmental factors (photodermatose) and potential complications arising from their location.

**Figure 2:**
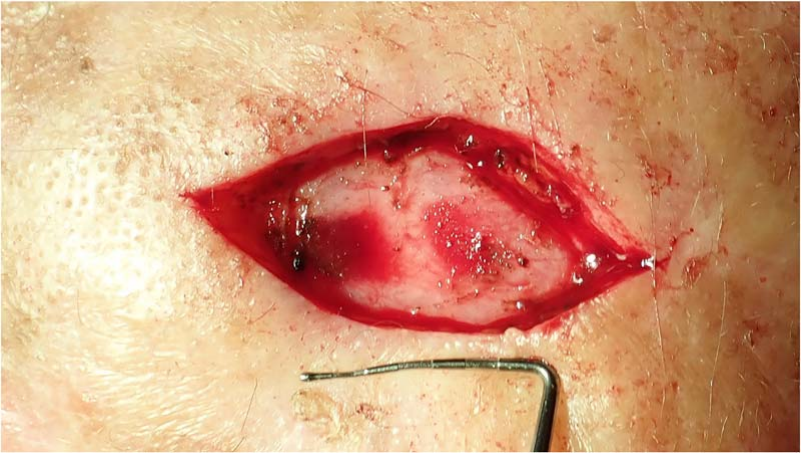
The excisional biopsy was undertaken to remove the entire lesion. The obtained tissue sample was then sent to the pathology laboratory for analysis. The defect is too large to enable primary wound closure.

**Figure 3:**
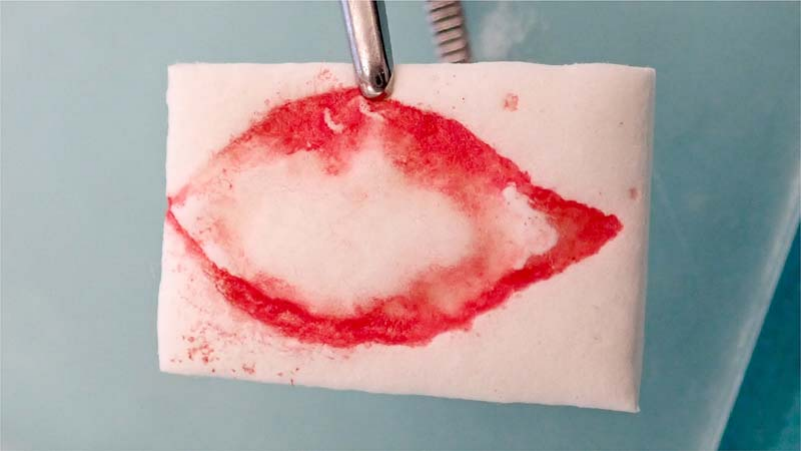
The excised area was marked on a resorbable collagen matrix (Mucograft®, Geistlich, Wolhusen) like an impression. This material was used to cover the defect and to avoid any donor site morbidity from creating flaps to close the defect.

**Figure 4:**
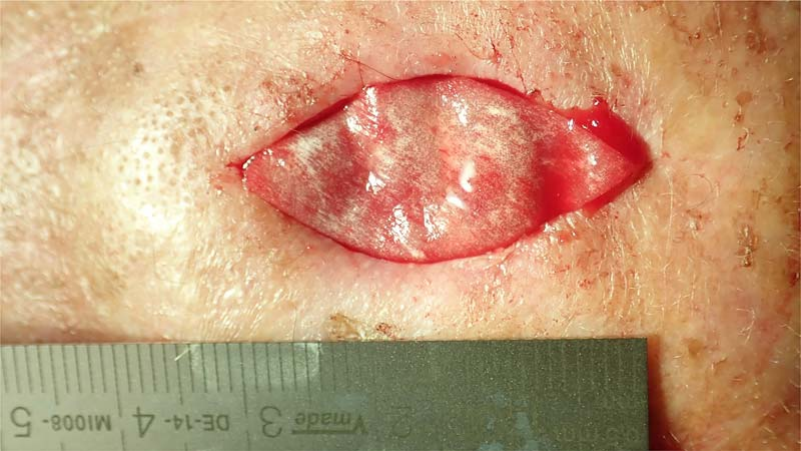
Fitting of the matrix.

**Figure 5:**
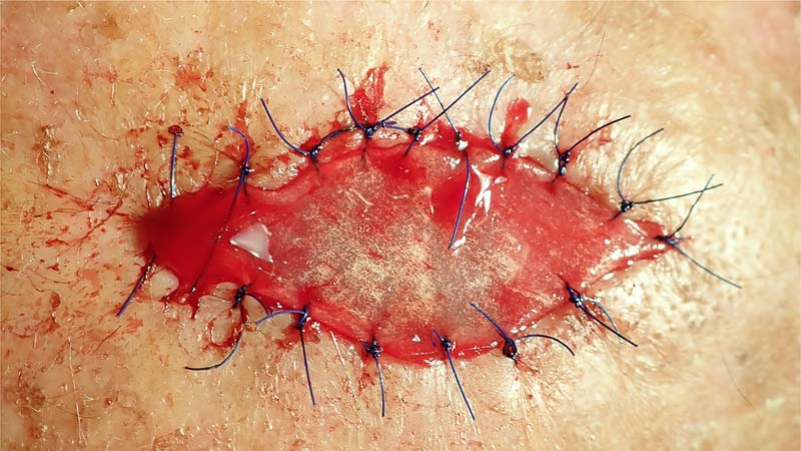
The matrix is fixed on the defect with 6.0 Sabafil sutures.

**Figure 6:**
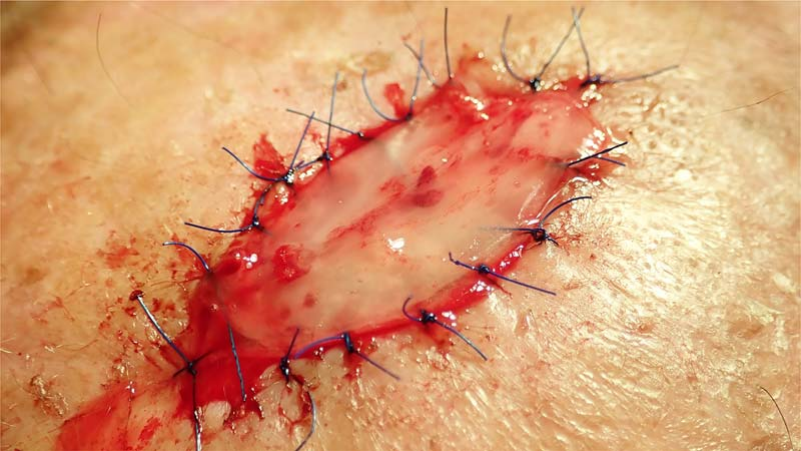
The collagen matrix is enriched with I-PRF. After centrifugation according to Coukroun’s protocol, I-PRF is derived from the patient’s blood. In this case, it is used to promote tissue healing.

**Figure 7:**
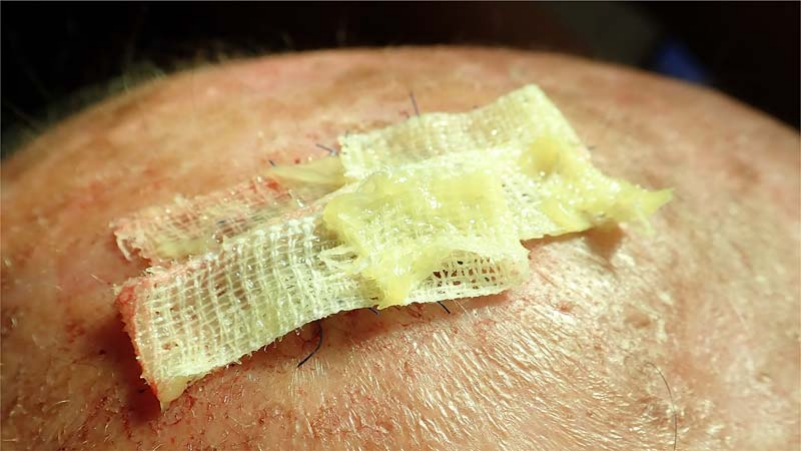
Stripes with Aureomycin (Chlortetracycline) are used to cover the defect. The patient was provided with postoperative care instructions, including wound care, hygiene, and the importance of follow-up appointments for monitoring and evaluation.

**Figure 8:**
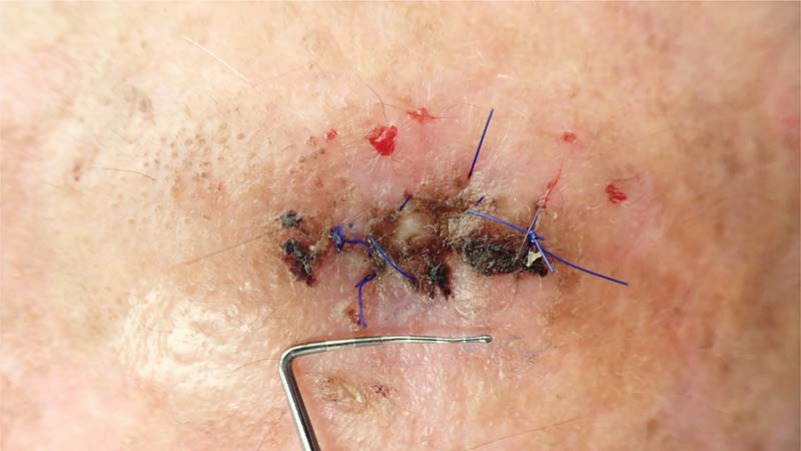
Clinical situation after 10 days; stitches will be removed at this appointment.

An 82-year-old Caucasian male presented with lesions on his neck and forehead, with recent changes in color and extension. Previously, he had undergone unsuccessful local steroid treatment for the lesions. Past medical history included arteriosclerosis with multiple stent implantations and diabetes (HbA1c > 8.5). His medication regimen comprised ASS 100 mg, Bisoprolol 12.5 mg, Ramipril 2.5 mg, Eplerenone 25 mg, Simvastatin 40 mg, Glimepiride 3 mg, Metformin 500 and Lantus 100 ml. The scalp lesion exhibited ulcerative and nodular features, raising suspicion for BCC (Fig. 1). Mohs excision was performed under local anesthesia (Ultracain DS Forte®, Septodont, Saint-Maur-des-Fossés, France; Fig. 2), followed by wound closure utilizing a resorbable collagen matrix (Mucograft, Geistlich, Basel, Switzerland; [7, 8]) and injectable platelet-rich fibrin (I-PRF) according to Choukroun’s protocol [9]. The hydrophilic collagen matrix, was placed on the defect and sutured without tension (Sabafil 6.0; Winsford, Cheshire, United Kingdom) [Figs 2–7]. Postoperative care instructions were provided, leading to a clinically healthy defect area at suture removal (day 10) and complete tissue coverage during wound healing [Fig. 8].

## Results

**Figure 9:**
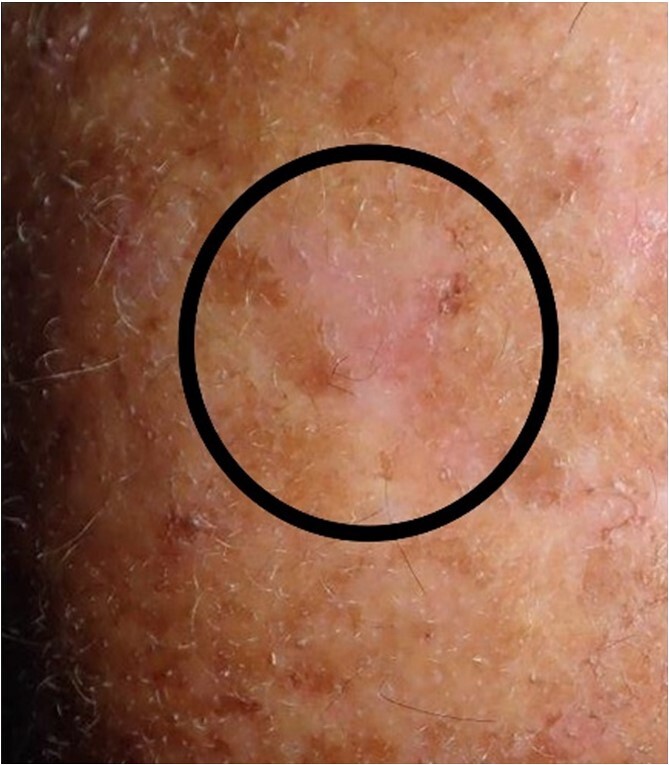
Situation 3 months after surgery. For patients with a large area of highly sun affected skin, it would be difficult to create a sufficient flap mobilization because of scars and to avoid camouflage of any lesions.

**Figure 10:**
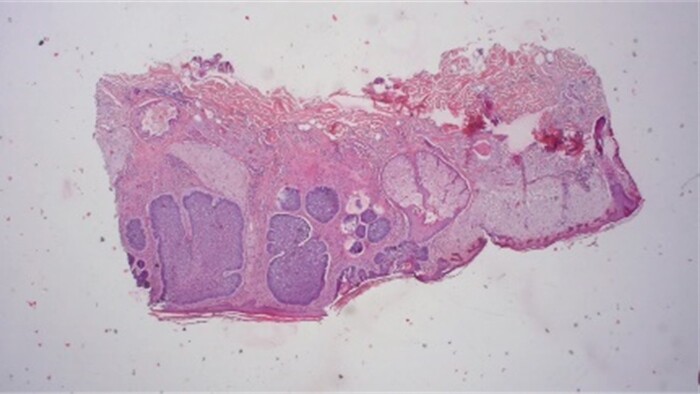
Histological results provide typically nesting patterns composed of proliferating basaloid cells. Basaloid cells arrange themselves in a palisading pattern at the periphery of the nests. This palisading is a key histological feature and is often linked to a picket fence appearance. The stroma surrounding the nests typically show variable degrees of fibrosis and inflammation. This fibrotic stroma may contain collagen fibers, and the inflammatory infiltrate may consist of lymphocytes and histiocytes (Dr.Jäkel, Pathologie Bremen, Bremen).

After a 3-month healing period, the area exhibited healthy skin without signs of cancer relapse [Fig. 9]. Immunohistological analysis post-Mohs excision confirmed the infiltrative growth of tumor cells, diagnosing BCC, which was excised with a safety range of at least 1 mm (Fig. 10).

## Discussion

Reconstruction of post-excisional defects on the scalp presents challenges due to limited skin availability, skin texture and the presence of hair follicles [10, 11]. In this instance, the patient’s baldness facilitated surgical preparation and planning, as well as post-surgical wound healing. Various surgical options are available for defect reconstruction, but due to the lack of evidence-based research, no guidelines exist for reconstruction methods and techniques, particularly for facial subsites [12]. A comprehensive understanding of general principles of reconstruction, aesthetic goals, flap vascularization and anatomy is imperative. Besides psychological parameters, issues such as wound care burden, itching, bleeding or public embarrassment should be included in evaluations and considered before deciding on surgical procedures. New strategies in wound healing aim to reduce healing time, improve aesthetic outcomes and minimize patient comorbidity.

Initially developed for bone regeneration, barrier membranes have been applied in periodontal regeneration and established as Guided Bone Regeneration to regenerate alveolar bone defects [13, 14]. A previous study demonstrated that the use of a collagen matrix after tumor resection in the head and neck area yielded comparable results to autologous skin grafts, as assessed by the Patient and Observer Scar Assessment Scale [15]. Other matrices, such as amniotic membrane based on collagen, have also shown promise in skin regeneration by providing growth factors through endogenous cells and facilitating tissue remodeling. The use of the collagen matrix, as demonstrated in this case, offers a new opportunity to enhance regeneration. By applying the matrix at the time of excision, the need for a second-stage surgery and the granulation phase typically associated with wound healing can be avoided. This may benefit patients with healing disorders or multimorbidity by reducing clinic visits and conserving resources. Additionally, by using a substitute, overlapping of areas and donor site morbidity can be minimized. The bilayer structure of the matrix allows for open healing and soft-tissue structure filling of the defect, which may further enhance regeneration.

## Conclusion

This case report illustrated that the usage of I-PRF in addition to a collagen matrix improved wound healing and reduced donor site morbidity. The matrix provided mechanical coverage of the wound and promoted soft tissue healing, while I-PRF bioactivated the matrix and surrounding tissues. In summary, I-PRF appears to offer benefits for several aesthetic indications, as assessed by patients themselves and evidenced by improved skin parameters.

## Data Availability

Data available on request due to privacy/ethical restrictions.
